# The Role of Dietary Glycemic Index and Glycemic Load in Mediating Genetic Susceptibility via MC4R s17782313 Genotypes to Affect Cardiometabolic Risk Factors among Apparently Healthy Obese Individuals

**DOI:** 10.1155/2022/3044545

**Published:** 2022-11-16

**Authors:** Mahdieh Khodarahmi, Goli Siri, Mohaddeseh Mohammadi, Mahdieh Abbasalizad Farhangi, Samira Aleseidi

**Affiliations:** ^1^Department of Community Nutrition, Faculty of Nutrition and Food Science, Tabriz University of Medical Sciences, Tabriz, Iran; ^2^Department of Internal Medicine, Amir-Alam Hospital, Tehran University of Medical Sciences, Tehran, Iran; ^3^Nutriton Research Center, Tabriz University of Medical Science, Tabriz, Iran; ^4^Drug Applied Research Center, Tabriz University of Medical Sciences, Tabriz, Iran; ^5^Rheumatology Research Center, Tehran University of Medical Sciences, Tehran, Iran

## Abstract

**Background:**

The association of genetic and dietary factors with occurrence and progression of chronic diseases such as metabolic syndrome (MetS) has long been addressed but there is a lack of evidence for complex interrelationships, including direct and indirect effects of these variables. Hence, this study is aimed at evaluating the mediating role of glycemic indices in the association of melanocortin-4 receptor (MC4R) rs17782313 polymorphism, sociodemographic, and psychological factors with the risk of MetS in obese adults using structural equation modeling.

**Methods:**

We performed a cross-sectional analysis of data from 287 apparently healthy adults. Dietary glycemic index (GI) and glycemic load (GL) were calculated from a validated 147-item food frequency questionnaire (FFQ). MC4R s17782313 genotypes were determined by polymerase chain reaction-restriction fragment length polymorphism (PCR-RFLP) method. Structural equation modeling was used to explore direct and indirect effects of genetic and nongenetic factors on MetS.

**Results:**

MC4R gene variant was directly associated with the risk of MetS (*B* = 0.010; *P* = 0.023). On the other hand, this variant was found to be indirectly and positively associated with LDL-C (*B* = 6.589; *P* = 0.042) through mediatory effects of GI and GL. Moreover, GI and GL also mediated indirect positive effects of sex and age on LDL-C (*B* = 3.970; *P* ≤ 0.01; *B* = 0.878; *P* ≤ 0.01, respectively) and HDL (*B* = 2.203; *P* ≤ 0.01; *B* = 0.129; *P* ≤ 0.01, respectively). MC4R rs17782313 polymorphism had positive effects on GI (*B* = 1.577; *P* ≤ 0.01) and GL (*B* = 1.235; *P* ≤ 0.01).

**Conclusion:**

Our data may state a hypothesis of the mediating effect of quantity and quality of carbohydrates consumed in relationship between genetic susceptibility to obesity and cardiometabolic risk factors. Further analyses should be carried out in high-quality cohort studies in order to confirm the findings.

## 1. Introduction

The world has witnessed a dramatic raise in the obesity incidence and its related mortality rate [1]. Obesity is a metabolic disorder and most important public health problem among both developed and developing countries and for all ages [2]. According to the results of a study conducted in 2017, prevalence of obesity was about 25% among Iranian adults [3]. Obesity is strongly related to a number of complications and pathologic conditions such as insulin resistance, hypertension, cardiovascular diseases (CVDs), and metabolic syndrome (MetS) [4]. The pathophysiology of obesity is complex and multifactorial that most often involves complex interactions between genetic and social-environmental factors, both of which may be connected with other risk factors [5].

Among environmental factors, dietary factors and socioeconomic status (SES) have been proposed as potential modifiable parameters in development of obesity and its related consequences such as MetS [6]. There is raising evidence which high carbohydrate diets affect features of MetS such as dyslipidemia and insulin resistance [7]. In this context, studies have extensively focused on refined grains and sugars [8]. Recently, comprehensive assessment of characterization of the diets has attracted a particular attention because they comprise a true image of usual diet [9]. The glycemic index (GI) concept, as one of metrics for carbohydrate quality, was introduced by Jenkins et al. for the first time, to provide comparison of the physiological effects of the carbohydrate of foods consumed. It is defined as the rate of glycemic response and demand for insulin after eating a meal [10]. Since GI only represents the quality of dietary carbohydrate, glycemic load (GL), which is a product of GI, was determined to account for real carbohydrate content [11]. Various researches have shown that there are relations between dietary GI, GL, cardio metabolic risk factors, and MetS [12–15]. However, there are some ambiguities regarding the clinical outcomes [16, 17]. Because of the cofounding effects of potential variables (especially sociodemographic variables), it is hard to evaluate the true association of dietary glycemic indices with obesity and its related cardiometabolic risk factors [18]. Moreover, some studies have shown that psychological factors through increasing unhealthy eating behaviors and sedentary lifestyle indirectly can lead to promote the progress of obesity [19, 20].

On the other hand, genetic factors can also contribute to the susceptibility to weight gain and its comorbidities. Efforts of genetic studies to find the genes involved in the energy balance have led to detection of several obesity-related genes including the leptin [21], melanocortin-4 receptor (MC4R) [22], and fat mass and obesity-associated (FTO) [23]. MC4R gene plays an essential role in the central control of energy homeostasis and development of obesity-associated metabolic diseases [24]. The rs17782313 which is the most well-known single-nucleotide polymorphism (SNP) of MC4R [25] has been mapped on chromosome18q 21.32 at 188 kb downstream of the gene, and it seems that this variant plays a strong regulatory role in function of this gene [26, 27]. Several studies have shown the C allele of rs17782313 is associated with weight gain and obesity-related complications such as glucose intolerance and hyperlipidemia [28–30]. For instance, Yang et al. have revealed that the CC genotype of the rs17782313 is related to higher serum TG levels [29], or Marcadenti et al. whose results have indicated the positive association of MC4R common variant (rs17782313) with the risk of type-2 diabetes mellitus [30]. However, some studies have found no association, and the results are ambiguous [22, 31]. It is worth noting that some discrepancies can be due to the role of diet, as a lifestyle factor, in modifying the influences of MC4R gene variations [32]. Totally, due to hidden reciprocal relationships and high collinearity that exist among life-style, psychological, and genetic factors, direct and indirect mechanisms underlying the relationship between genetic factors and obesity and its-related metabolic outcomes such as MetS remain controversial. Hence, according to aforementioned, in order to evaluate the role of these variables in incidence of MetS and obesity-related cardio-metabolic traits, it is needed to examine the complex and interrelated pathways instead of studying a single relationship.

Structural equation modeling (SEM) is a multivariate alternative technique for the concurrent studying of complex relationships and latent reciprocal effects between variables by using several regressions and path analyses simultaneously [33]. Therefore, the current study was designed to investigate the mediating effects of glycemic indices in the association of psychological parameters, sociodemographic factors, and genetic susceptibility to obesity with MetS and cardiometabolic risk factors among obese population using SEM method.

## 2. Methods and Material

### 2.1. Demographic Characteristics and Design

This study was conducted between November 2017 and October 2018 in Tabriz, the capital city of East Azerbaijan province in the northwest of Iran. 287 apparently healthy obese subjects (147 men and 140 women) were recruited in this cross-sectional analysis by convenience sampling method through posters and flyers placed in hospitals and public areas. We followed the methods of Khodarahmi et al. in our project [34]. Participants were included according to the following criteria: age 20–50, being obese (BMI ≥ 30 kg/m^2^). In the beginning, a total of 350 subjects were willing to be screened for participation in the study. After screening for eligibility according to the inclusion and exclusion criteria, 63 individuals were excluded from the study. Exclusion criteria were as follow: pregnancy, lactation or menopausal, having diabetes, hypertension, hyperlipidemia, hepatic disorders, cardiovascular and renal diseases, or having any recent surgery such as bariatric. As well as individuals taking any medications and supplements effective on weight and variables studied (loop diuretics, corticosteroids or antidepressants, and antihypertensive agents) were excluded. Eventually, after applying these eligibility criteria, 287 subjects were included in analyses. By considering maximum RMSEA of 0.08 [35], *α* = 0.05, and power of 80%, with the use of statistica software (version 10), the minimum sample size was estimated at 184. Overall, 278 subjects who agreed to participate were evaluated in the present research. The study was approved by the Ethical Committee of the Tabriz University of Medical Sciences (registration code IR.TBZMED.REC.1398.460 and IR.TBZMED.REC.1396.768), and written informed consents were obtained from all subjects prior to participation in the study.

According to the National Cholesterol Education Program (NCEP) Adult Treatment Panel (ATP) III definition, metabolic syndrome was identified if three or more of the following criteria were met: fasting blood sugar ≥ 100 mg/dl, waist circumference ≥ 102 cm (men) or ≥88 cm (women), systolic/diastolic blood pressure ≥ 130/85 mmHg, fasting triglyceride (TG) level ≥ 150 mg/dl, and fasting high-density lipoprotein (HDL) cholesterol level less than 40 mg/dl (men) or 50 mg/dl (women) [36].

### 2.2. Dietary Intake Assessment, Appetite Measuring, and Calculation of Dietary GI and GL Values

A reliable and validated 147-item food frequency questionnaire (FFQ), which its validity and reliability had been confirmed in Iran, was applied to determine the usual dietary intake of participants [37, 38]. Dietary data were gathered through face-to-face interviews by trained dietitians. Individuals were asked to determine the frequency and amount of the intake of each given food item based on a daily, weekly, monthly, and yearly basis during the previous year, and, subsequently, the portion sizes of the consumed foods were converted to grams by household measurements. Iranian Food Composition Table (FCT) was used to evaluate daily energy and nutrient intakes [39] and complemented by United States Department of Agriculture FCT [40]. Of the 147 food and beverage items which were included in the FFQ, 100 items contain available carbohydrate. The value of dietary GI for the major carbohydrate-containing foods was derived from national references and as the Iranian food table of GI is incomplete, International GI table was used for the unrecorded food items [41–43]. Total dietary GI was estimated by using the following formula: ∑(GI_a_ × available carbohydrate_a_)/total available carbohydrate [44]. In the above formula, available carbohydrate was calculated as total carbohydrate_a_ minus dietary fiber_a_, and, accordingly, glucose was considered as reference. Dietary GL was determined based on the following formula: (total GI × total available carbohydrate)/100 [44]. Then, participants were categorized into tertiles of dietary GI (T1: ≤67.20, T2: 67.21-72.35, T3: ≥72.36) and GL (T1: ≤161.53, T2: 161.54-219.42, T3: ≥219.43).

Appetite was assessed by means of a 10 cm visual analog scale (VAS) questionnaire which was validated in previous studies [45]. This questionnaire contains six questions about sensation of prospective food intake, hunger, satiation, and the desire to eat something sweet, salty, or fat [45]. The responses were measured by make a mark on a 10 cm straight line for each question, and quantification of each VAS score was carried out by measuring the distance from the left side of the line to the mark.

### 2.3. Assessment of Sociodemographic Anthropometric Variables and Blood Pressure Measurements

All information was collected by a trained interviewer. Socioeconomic status was evaluated by the questions on occupation, educational status, family size, and home ownership as individual indicators and, then, the total score was calculated and participants were classified into three categories: low, middle, and high based on SES tertiles. Using a short form of the International Physical Activity Questionnaire, physical activity was assessed [46]. Anthropometric measurements were done by an expert using standardized methods and equipment. Height and weight were measured while the participant stood in light clothing and in bare foot with the use of a tape measure and Seca scale (Seca, Germany) with accuracy of 0.1 cm and 100 g, respectively. The BMI of the participants was calculated as weight (kg) divided by height squared (m^2^). The waist circumference (WC) was measured at the narrowest level and at the end of normal exhalation by a flexible inelastic tape to the nearest 0.1 cm. Blood pressure (BP) measurements were carried out using a standardized mercury sphygmomanometer twice after a 15 min rest in a sitting position. The mean of the two measurements was considered as the participant's BP.

### 2.4. The Mental Health Assessment

Mental health was assessed by the valid and reliable Depression, Anxiety and Stress Scale-21 Items (DASS-21) questionnaire, which consists of three self-report subscales including depression, anxiety, and stress [47, 48]. The Cranach's *α* coefficient for this questionnaire among Iranians has been reported as 0.77, 0.79, and 0.78 for depression, anxiety, and stress, respectively [47]. This questionnaire comprises three categories of 7-item self-report scale (depression, anxiety, and stress), and the responses are rated using a 4-point Likert scale from 0 to 3. For each subscale, total score was estimated by summing the scores for the relevant questions and multiplying them by 2 which could range from 0 to 42. Then, individuals were classified into 5 categories: normal, mild, moderate, severe, and extremely severe [49]. Indeed, higher scores indicate greater severity of psychological disorders.

### 2.5. Measurement of Biochemical Parameters

Blood samples were drawn from participants after an overnight fasting period. To separate serum, blood samples were centrifuged at 4500 rpm, for 10 min at 4°C, and then extracted serum was stored (-80°C) until assay. In order to evaluate levels of fasting serum glucose, TG, total cholesterol (TC), and HDL-cholesterol, commercially available enzymatic kits (Pars Azmoon, Tehran, Iran) were used. Low-density lipoprotein-cholesterol (LDL-C) concentration was determined by Friedewald equation [50]. An enzyme-linked immunosorbent assay (ELISA) (Bioassay Technology Laboratory, Shanghai Korean Biotech, and Shanghai City, China) was used to measure serum insulin concentrations. For evaluating insulin sensitivity, the homeostasis model assessment of insulin resistance (HOMA-IR) and quantitative insulin sensitivity check index (QUICKI) were calculated based on standard formulas [51, 52]. Atherogenic index of plasma (AIP) was calculated as Lg_10_ (serum triglycerides/serum HDL-cholesterol) [53].

### 2.6. Genetic Analysis

Genomic DNA was extracted from 5 ml peripheral whole blood sample by using a standard phenol/chloroform technique [54]. The MC4R rs17782313 polymorphism was genotyped by polymerase chain reaction-restricted length polymorphism (PCR–RFLP) analysis as follows. DNA fragment containing MC4R variant was amplified by the forward primer 5′ AAG TTC TAC CTA CCA TGT TCT TGG 3′ and reverse primer 5′ TTC CCC CTG AAG CTT TTC TTGTCA TTT TGA T 3′ (Macro-gene, Korea). PCR was carried out under the following conditions: initial denaturation at 95°C for 2 min, followed by 35 cycles of denaturation (95°C for 30 s), annealing (58°C for 30 s), and extension (72°C for 30 s), with the final extension at 72°C for 5 min. PCR amplification was performed in a final volume of 20 *μ*l containing 10 *μ*l of Taq DNA Polymerase 2× MasterMix (Ampliqon; Germany), 200 ng of DNA, 0.5 *μ*mol of each primer, and 9 *μ*l of distilled water. Then, 7 *μ*l of PCR product containing the rs17782313 polymorphism was digested with 0.5 *μ*l of BclI (10 U/*μ*l) restriction enzyme (Fermentas, Germany) and 2 *μ*l of 10 × restrictions G-buffer at 56°C overnight. The electrophoresis on 2% agarose gel was conducted, and DNA fragments were visualized on a Gel Doc-system (U.V.P Company, Cambridge, UK). After that, the C allele was distinguished as fragments with length of 137 bp (uncut product), and the T allele was detected as cut 107 and 30 bp fragments.

### 2.7. Statistical Analyses

In statistical analysis, data were analyzed using the SPSS software version 23.0 (SPSS Inc., Chicago, IL, USA), and *P* values < 0.05 were considered as statistically significant. Normality distribution of continuous variables was examined by the Kolmogorov-Smirnov test. Descriptive analyses were expressed as mean ± SD for continuous variables with normal distribution, frequencies, or percentages for categorical variables and median (25th and 75th percentile) for those with a skewed distribution. Participants were categorized based on tertiles cut-off points of dietary GI and GL. Quantitative and qualitative variables were compared across tertiles using one-way analysis of variance (ANOVA) and chi-square tests, respectively. SEM is a multivariate statistical technique that often consists of two important stages, the measurement model and the structural model (direct and indirect pathways of associations between latent and other observed variables) [33]. In this dataset, measurement model (unobserved or latent constructs identified using factor analysis) was not applicable.

**Figure 1:**
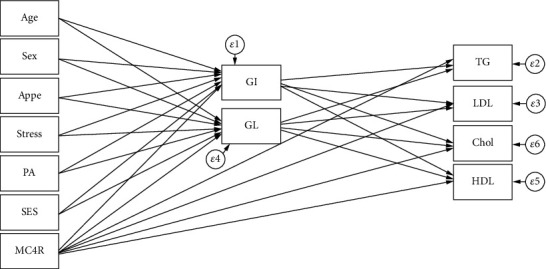
Hypothesized models in which GI and GL as mediating variables relate MC4R rs17782313 polymorphism, sociodemographic, and psychological parameters to serum lipids. Abbreviations: MC4R: melanocortin-4 receptor; GI: glycemic index; GL: glycemic load; SES: socioeconomic status; PA: physical activity; Appe: appetite; LDL-C: low-density lipoprotein cholesterol; HDL: high-density lipoprotein; TG: triglyceride; Chol: cholesterol.

**Figure 2:**
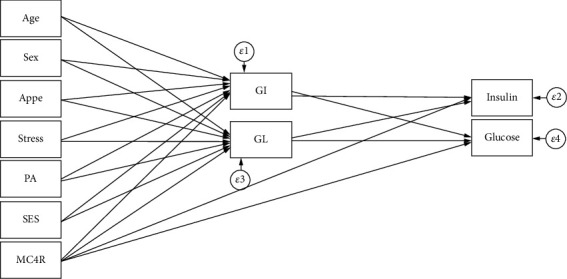
Hypothesized models in which GI and GL as mediating variables relate MC4R rs17782313 polymorphism, sociodemographic, and psychological parameters to serum glycemic levels. Abbreviations: MC4R: melanocortin-4 receptor; GI: glycemic index; GL: glycemic load; SES: socioeconomic status; PA: physical activity; Appe: appetite.

**Figure 3:**
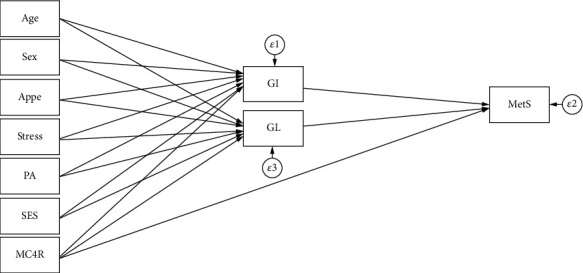
Hypothesized models in which GI and GL as mediating variables relate MC4R rs17782313 polymorphism, sociodemographic, and psychological parameters to MetS. Abbreviations: MC4R: melanocortin-4 receptor; GI: glycemic index; GL: glycemic load; SES: socioeconomic status; PA: physical activity; Appe: appetite; MetS: metabolic syndrome.

SEM analysis was carried out to test the proposed conceptual models which were identified according to previous studies and logical grounds, the mediating effects of dietary glycemic indicators on the role of genetic susceptibility, sociodemographic variables, and mental health in MetS risk and metabolic risk factors as well (shown in Figures 1–3). In the present study, several path analyses (regression analysis) were run to identify 3 following purposes: (1) the association of sociodemographic, mental characteristics, and genetic factors with cardiometabolic risk factors is mediated by quantity and quality of carbohydrates consumed and (2) the associations between all the aforementioned variables and MetS risk are mediated by these glycemic indicators. Model estimates were made by maximum likelihood estimation method. Fitting of conceptual models to the data was assessed using the usual goodness of fit indices including the comparative fit index (CFI) > 0.90 [55], standardized root mean square residual (SRMR) < 0.08 [35], chi-square test (*χ*^2^/degrees of freedom (df)) ratio < 5 [56], and root mean square error of approximation (RMSEA) ≤ 0.08 [35]. All analyses were conducted using STATA version 14.2 and Mplus software (version 7.4; Muthén and Muthén).

## 3. Results

**Figure 4:**
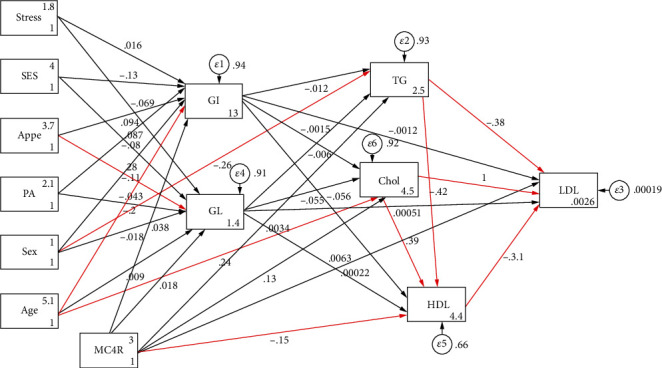
Path analysis diagram with standardized estimates illustrating the total effects of MC4R rs17782313 polymorphism, diet, sociodemographic, and psychological parameters on lipid profile among obese adults. Abbreviations: MC4R: melanocortin-4 receptor; GI: glycemic index; GL: glycemic load; SES: socioeconomic status; PA: physical activity; Appe: appetite; LDL-C: low-density lipoprotein cholesterol; HDL: high-density lipoprotein; TG: triglyceride; Chol: cholesterol. ^∗^All path coefficients are standardized. Red arrows mean *P* value ≤ 0.05. ^£^Total effect is defined as the sum of direct and indirect effects.

**Figure 5:**
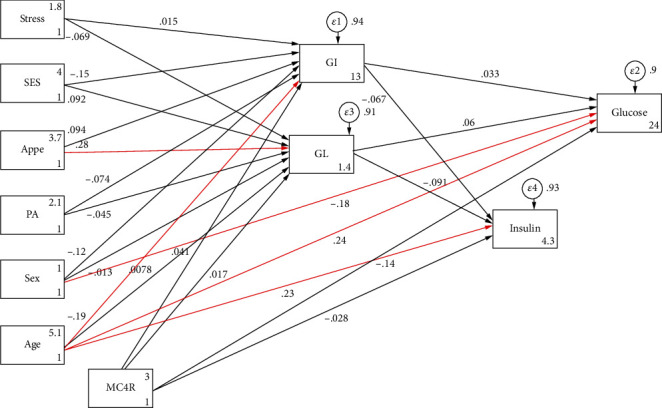
Path analysis diagram with standardized estimates illustrating the total effects of MC4R rs17782313 polymorphism, diet, sociodemographic, and psychological parameters on serum glycemic levels among obese adults. Abbreviations: MC4R: melanocortin-4 receptor; GI: glycemic index; GL: glycemic load; SES: socioeconomic status; PA: physical activity; Appe: appetite. ^∗^All path coefficients are standardized. Red arrows mean *P* value ≤ 0.05. ^£^Total effect is defined as the sum of direct and indirect effects.

**Figure 6:**
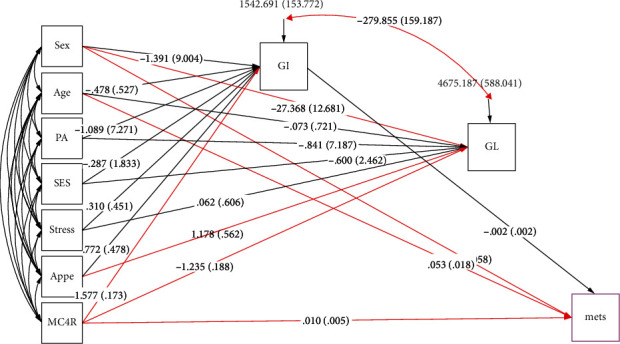
Structural equation model diagram with standardized estimates for total effects of genetic, sociodemographic, and psychological parameters and diet on metabolic syndrome among obese adults. Abbreviations: MC4R: melanocortin-4 receptor; GI: glycemic index; GL: glycemic load; SES: socioeconomic status; MetS: metabolic syndrome; PA: physical activity; Appe: appetite. ^∗^All path coefficients are standardized. Red arrows mean *P* value ≤ 0.05. ^£^Total effect is defined as the sum of direct and indirect effects.

Characteristics of the participants (sociodemographic, genetic, psychological, and metabolic parameters), according to the GI and GL tertiles among male and female subjects, are shown in Tables 1 and 2, respectively. There were no significant differences in the mean values of anthropometric, sociodemographic, and mental health variables across tertiles of dietary GI and GL in both women and men. GI was positively associated with high LDL-C levels in men (*P* = 0.024), and, similarly, a higher dietary GL intake was significantly related to the higher LDL-C (*P* = 0.050) and cholesterol (*P* = 0.022) concentrations in women. Additionally, there were statistically significant differences in genotype frequencies of the MC4R rs17782313 polymorphism across tertiles of GI in both women (*P* = 0.044) and men (*P* = 0.011). The direct and indirect pathways of the association between study variables (genetic, dietary, sociodemographic, and psychological variables) and serum lipid profile (model 1) and serum glycemic levels (model 2) among obese individuals were assessed using SEM, and significant results are summarized in Table 3. A significant negative direct effect on HDL levels was found for the MC4R rs17782313 polymorphism (*B* = −1.880; *P* = 0.029), and, on the other hand, this variant was found to be indirectly and positively associated with LDL-C (*B* = 6.589; *P* = 0.042) through mediatory effects of GI and GL. GI and GL also mediated indirect positive effects of sex and age on LDL-C (*B* = 3.970; *P* ≤ 0.01; *B* = 0.878; *P* ≤ 0.01, respectively) and HDL (*B* = 2.203; *P* ≤ 0.01; *B* = 0.129; *P* ≤ 0.01, respectively). In model 2, the direct relationships between age (*B* = 0.003; *P* ≤ 0.01) and sex (*B* = −0.028; *P* = 0.029) and serum glucose levels were found. In addition, age was directly associated with insulin concentrations (*B* = 0.008; *P* ≤ 0.01). However, no significant indirect relationship was found in this model (Table 3). The goodness of fit indices for models 1 and 2 indicated an acceptable fit (*χ*^2^/d.f. = 1.207; RMSEA = 0.038 (95%CI = 0.000, 0.082); CFI = 0.996 and *χ*^2^/d.f. = 1.030; RMSEA = 0.015 (95%CI = 0.000, 0.091); CFI = 0.987, respectively) (Table 4). Path analysis diagrams with standardized estimates investigating total effects of study variables on serum lipid profile and glycemic levels are depicted in Figures 4 and 5, respectively. The third model (Table 3) was tested to explore the direct and indirect associations between genetic, sociodemographic, and psychological parameters and MetS risk, and its results are illustrated in Figure 6; its goodness-of-fit indices showed an acceptable fit (*χ*^2^/d.f. = 1.203; RMSEA = 0.037 (95%CI = 0.000, 0.133); CFI = 0.994). The results showed that the associations of mentioned variables with MetS were not indirect, but instead were direct. MC4R gene variant was positively associated with the risk of MetS (*B* = 0.010; *P* = 0.023). In addition to the direct effect of age on MetS (*B* = 0.053; *P* ≤ 0.01), sex had also a direct negative relationship with MetS (*B* = −0.605; *P* = 0.024). The standardized estimates illustrating the total effects of genetic, sociodemographic, and psychological parameters and diet on MetS risk are shown in Table 5. MC4R rs17782313 polymorphism had positive effects on MetS risk (*B* = 0.010; *P* = 0.023), GI (*B* = 1.577; *P* ≤ 0.01), and GL (*B* = 1.235; *P* ≤ 0.01). On the other hand, appetite was a significant predictor of GL (*B* = 1.178; *P* = 0.018).

## 4. Discussion

The present study is the first, to the best of our knowledge, to test direct and indirect effects of genetic, psychological, and modifiable risk factors on cardiometabolic risk factors and MetS risk among obese subjects using structural-equation modeling. Our study provided scientific evidence of indirect effects of near MC4R rs17782313 polymorphism through the mediation of dietary glycemic indices on some of cardiometabolic risk factors such as LDL-C levels. However, the effect of the rs17782313 variant on MetS risk was not mediated via these dietary indices, and only direct positive associations between this polymorphism and MetS were found. Another main finding in this research was the negative direct association of MC4R variant with serum HDL concentrations. Likewise, we observed significant direct paths from the age and gender to some lipid profile (triglyceride and cholesterol), serum glycemic levels (glucose and insulin), and MetS. Moreover, we found that compared with those (both female and male subjects) in the first tertile, participants in top tertile of dietary GI were more likely to have CC genotypes.

Diet as a first-line intervention in the prevention and treatment of MetS, diabetes, and CVD risk factors has been gaining attention [57]. In this regard, GI and GL which take into account both quality and quantity of the dietary carbohydrate are of high priority and can contribute to nutritional therapy for chronic diseases. Although we did not find any evidence for mediation effect of dietary GI and GL on MetS, several previous meta-analyses of randomized controlled trials (RCTs) reported that low-GI or GL diets than control diets resulted in lower cardiovascular risk factors such as serum glucose, total cholesterol, and LDL-C [58, 59]. On the other hand, some meta-analyses have not confirmed such results [60]. Moreover, results from prospective studies in term of GI or GL-MetS association are inconsistent [61]. These varied results could be, in part, explained by the differences in the sample size, study design, and subject's characteristics such as genetic structures.

Regarding the genetic determinants, near MC4R rs17782313 was found to be directly related to MetS risk in the current research while the indirect association of this variant with MetS through dietary glycemic indicators was not shown. Our observations were in line with earlier evidence that revealed a significant association between near MC4R rs17782313 and metabolic syndrome [62]. Additionally, accumulating epidemiological studies have reported the relationships of this polymorphism (rs17782313) with insulin resistance, type 2 diabetes, and also some component of metabolic syndrome [63, 64]. Accordingly, consistent with abovementioned studies, a significant direct association was revealed between rs17782313 and serum LDL-C and HDL in the present study. Noticeably, indirect positive association of this variant with LDL-C in our study suggests that both quality and quantity of the carbohydrate ingested are a mechanism by which this obesity-susceptibility gene may influence obesity and its-related cardiovascular risk factors and, so, they must be targeted in treatment of obesity and other chronic diseases. Although the biological mechanism of the relationship between rs17782313 and risk of MetS and its components are not exactly clear and require to be investigated in further studies, animal studies have reported that MC4R knockout mice exhibit hepatic insulin resistance and display leptin resistance [65] and, also, an increase in lipid uptake, triglyceride synthesis, and fat accumulation in white adipose tissue was seen [66]. In other words, as the MC4R gene is highly expressed in the central nervous system plays an important role as a leptin-targeted neural circuit in controlling feeding behavior and energy expenditure [67]. Both human and animal studies have suggested that the association between MC4R rs17782313 and MetS is at least partially independent of body weight [68, 69], and, consequently, insulin resistance may mediate part of the relationship of MC4R rs17782313 with MetS. Nevertheless, further studies are required to clarify the potential biological pathways by which MC4R rs17782313 influences the risk of MetS.

As mentioned previously, the associations of psychological, dietary, genetic, and sociodemographical factors with MetS and cardiometabolic risk factors have been mostly assessed by ANOVA or traditional regression methods [64, 70], and there is no study that has proposed and tested these variables under a conceptual model (i.e., SEM and with examining indirect effects of a set of variables) that makes it difficult to compare and discuss our findings with those of other studies. Nevertheless, there were several investigations that have reported the independent associations of the MC4R rs17782313 polymorphism and indicators of dietary carbohydrate quality (GI and GL) with MetS risk [62, 70, 71]. For instance, several observational studies have evaluated the relationships of these dietary indicators with MetS and blood lipids, and in most of them, higher dietary GI or GL scores were related to an increased prevalence of MetS [61, 70] and blood lipid disturbances [61]. Nevertheless, contrary to expectations, the outcomes of the present investigation showed no significant relationship between GI or GL and the presence of MetS which this result is consistent with what other studies have reported regarding this relation [13]. On the other hand, univariate analysis in our research revealed a positive association between dietary GI and LDL-C in men, and a similar association was observed as well, in relation to GL in women. These results are in agreement with some trial studies in which low-GI diets decreased TG, LDL cholesterol, and the total to HDL cholesterol ratio [72]. Despite the fact that pathways linking GI and GL to dyslipidemia are largely unknown, it seems that high-GI/GL diets, which cause a greater postprandial increase in insulin levels, may lead to the development of dyslipidemia through an increase in appetite, overeating, higher fat storage, and further release of free fatty acids [73]. Besides, the present research found that the effects of age and gender on the serum lipid concentrations are partly mediated through diets with high GI and GL which suggests that they can be targeted in clinical practice. Likewise, direct associations were observed between these factors (age and gender) and serum glycemic levels in our analysis. These observations were in agreement with earlier studies that indicated gender significantly modified the effects of GI and GL on cardiometabolic risk factors. In this regard, a systematic review and meta-analysis of 15 prospective cohort studies indicated that high glycemic load score was related to the higher risk of CVDs in women, but not in men [74]. Another finding of this study was that mutant homozygote genotype (CC) was significantly associated with a higher dietary GI score in both female and male subjects. Although there is no human study on the relationship between GI or GL and MC4R polymorphisms to compare accurately our observations, some studies are contrary to our finding, and they have demonstrated that this variant is associated with lower carbohydrate and protein intakes [75, 76]. The discrepancy in study characteristics and dietary assessment methods may explain these differences.

The results of our study should be interpreted in light of several potential limitations. First, owing to the cross-sectional nature of this study, causal relationships cannot be inferred, but it is useful for generating a hypothesis that can then be assessed by prospective studies. Second, as SEM analyses are highly dependent on the sample size and the scale of this study was relatively small, our findings should be taken with caution, and large longitudinal studies are required to confirm these results. Third, since this project was carried out in Tabriz with different dietary intakes and other various lifestyle factors, it is difficult to generalize the results of this study to all Iranian population. Fourth, it has been shown that obese subjects are more likely to underreport their dietary intakes and this phenomenon will bias diet-disease relationships [77]. Nevertheless, we excluded upper and lower extreme values of energy intake from the analysis to avoid this substantial error in this study. Fifth, despite adjustment for several confounders, residual confounding by unknown factors could not be fully eliminated. At last, since Iranian food glycemic index table contains only some limited food items, reference GI data from other countries were applied which might lead to an error in dietary GI and GL calculations. In spite of these limitations, several strengths need to be outlined. To the best of our knowledge, this is the first study investigating the mediating effects of glycemic indices in the association of psychological parameters, sociodemographic factors, and genetic susceptibility to obesity with MetS among obese population using a SEM method. SEM approach which can effectively control for measurement errors simultaneously investigates direct and indirect effects of a set of variables on a collection of outcomes. Other strengths of the study were the use of a reliable and validated FFQ to assess dietary intake.

In summary, our data may state a hypothesis of the mediating effect of quantity and quality of carbohydrates consumed in relationship between genetic susceptibility to obesity and cardiometabolic risk factors. Additionally, MC4R rs17782313 polymorphism had a positive and negative direct association with MetS risk and serum HDL concentrations, respectively. On the other hand, some of demographic factors in addition to direct effects could indirectly influence cardiometabolic risk factors, through mediation effects of dietary glycemic indices. Thus, it seems that focusing on improving the quality of carbohydrate particularly in individuals having a high genetic susceptibility to obesity would be useful for the prevention and control of obesity-related metabolic disorders. Although further analysis should be carried out in large-scale and prospective clinical trials to confirm these findings, low GI or GL diets may be one of a number of dietary modifications that help prevent and manage all or the main components of MetS.

## Figures and Tables

**Table 1:** Sociodemographic and anthropometric characteristics and cardiometabolic risk factors according to the tertiles of dietary glycemic indices in men.

	Glycemic index				Glycemic load			
	T1	T2	T3	P^∗^	T1	T2	T3	P^∗^
Age (y)	39.25 (5.76)	39.03 (7.48)	36.91 (6.37)	0.387	39.96 (6.31)	36.86 (6.80)	38.65 (6.42)	0.234
WC	108.75 (9.29)	114.72 (5.81)	112.54 (6.94)	0.475	112.39 (5.29)	114.16 (8.92)	112.69 (7.05)	0.588
BMI (kg/m^2)^	33.57 (4.06)	34.08 (2.53)	33.73 (2.86)	0.708	33.19 (2.59)	34.11 (3.67)	33.87 (2.96)	0.559
Physical activity level, (%)				0.207				0.461
Low	41.2	29.4	29.4		20.6	47.1	32.4	
Moderate	28.1	25.0	46.9		28.1	34.4	37.5	
High	20.0	46.7	33.3		23.3	26.7	50.0	
Marital status, (%)				0.479				0.554
Married	20.0	20.0	40.0		13.3	46.7	40.0	
Single	32.1	32.1	35.8		25.9	34.6	39.5	
SES, *n* (%)				0.450				0.468
Low	0.0	0.0	0.0		0.0	0.0	0.0	
Middle	16.7	50.0	33.3		26.7	23.3	50.0	
High	35.3	26.2	38.5		23.1	43.1	33.8	
Stress, *n* (%)				0.846				0.171
Normal	30.4	37.0	32.6		19.6	45.7	34.8	
Mild	29.4	35.3	35.3		47.1	23.5	29.4	
Moderate	29.4	23.5	47.1		35.3	17.6	47.1	
Severe	20.0	40.0	40.0		0.0	30.0	70.0	
Extremely severe	50.0	16.7	33.3		0.0	66.7	33.3	
Appetite	35.14 (9.29)	34.83 (9.94)	34.80 (9.73)	0.883	31.04 (9.67)	35.89 (9.72)	36.53 (8.84)	0.110
LDL-C, (mg/dl)	112.60 (25.50)^∗^	118.12 (24.27)	129.12 (29.34)^∗^	0.024	119.20 (27.43)	125.14 (28.04)	116.94 (26.57)	0.221
HDL, (mg/dl)	42.43 (7.50)	42.90 (8.69)	42.57 (7.62)	0.986	41.52 (7.75)	43.89 (8.93)	42.09 (6.71)	0.474
Cholesterol, (mg/dl)	185.21 (33.07)	184.28 (26.74)	197.86 (31.71)	0.084	184.00 (31.07)	197.69 (31.65)	185.41 (29.44)	0.095
TG, (mg/dl)	125.50 (96.50, 177.50)	116.00 (87.50, 134.50)	111.00 (78.00, 169.00)	0.214	90.00 (80.00, 134.00)	121.00 (92.00, 159.00)	123.00 (87.50, 169.00)	0.204
AIP	0.14 (0.23)	0.05 (0.23)	0.08 (0.27)	0.229	0.04 (0.26)	0.11 (0.25)	0.10 (0.23)	0.511
Glucose, (mg/dl)	92.00 (85.00, 99.25)	91.00 (86.50, 100.00)	91.00 (85.00, 101.00)	0.985	91.00 (85.00, 100.00)	92.00 (89.00, 101.00)	91.50 (84.00, 101.00)	0.844
Insulin, U/mL	15.30 (9.15, 26.60)	10.60 (8.05, 18.20)	11.50 (9.00, 17.20)	0.170	12.20 (10.00, 23.10)	13.20 (8.60, 23.60)	10.80 (8.75, 18.32)	0.473
HOMA-IR	3.58 (2.03, 5.97)	2.68 (1.75, 4.25)	2.70 (1.94, 4.00)	0.267	3.22 (2.10, 5.25)	3.20 (1.95, 4.88)	2.46 (1.86, 3.94)	0.452
QUICKI	0.32 (0.03)	0.33 (0.03)	0.33 (0.03)	0.371	0.32 (0.03)	0.32 (0.03)	0.33 (0.03)	0.493
SBP(mmHg)	112.21 (22.87)	117.93 (13.79)	118.71 (14.11)	0.286	117.17 (12.78)	120.29 (12.66)	112.12 (22.53)	0.217
DBP(mmHg)	71.75 (16.08)	78.10 (9.77)	77.14 (11.90)	0.108	77.61 (13.56)	76.71 (10.64)	73.65 (14.56)	0.486
MetS (%)	32.4	29.7	37.8	0.936	24.3	35.1	40.5	0.953
MC4R (%)				0.011				0.927
CC	27.3	18.2	54.5		27.2	36.4	36.4	
CT	26.1	34.8	39.1		21.7	43.5	34.8	
TT	40.0	51.4	8.6		25.7	40.0	34.3	

Data are presented as mean (SD) or median (25 and 75 percentiles). ^∗^Analysis of variance for continuous variables and *χ*^2^ test for categorical variables. Abbreviations: BMI: body mass index; WC: waist circumference; SES: socioeconomic status; HOMA-IR: homeostasis model assessment of insulin resistance; LDL-C: low-density lipoprotein cholesterol; HDL: high-density lipoprotein; SBP: systolic blood pressure; DBP: diastolic blood pressure; TG: triglyceride; QUICKI: quantitative insulin sensitivity check index; AIP: atherogenic index of plasma; MC4R: melanocortin-4 receptor.

**Table 2:** Sociodemographic and anthropometric characteristics and cardiometabolic risk factors according to the tertiles of dietary glycemic indices in women.

	Glycemic index				Glycemic load			
	T1	T2	T3	P^∗^	T1	T2	T3	P^∗^
Age (y)	38.85 (8.52)	38.13 (7.93)	36.81 (8.29)	0.570	38.90 (8.43)	36.57 (5.76)	38.08 (9.96)	0.477
WC	104.11 (11.39)	103.64 (10.46)	105.81 (8.09)	0.805	102.07 (9.60)	105.30 (11.40)	107.40 (8.95)	0.113
BMI (kg/m^2^)	35.36 (4.59)	35.94 (4.28)	35.72 (3.91)	0.865	35.07 (4.36)	36.19 (4.47)	36.04 (3.87)	0.570
Physical activity level, *n* (%)				0.668				0.467
Low	32.1	39.3	28.6		37.5	30.4	32.1	
Moderate	50.0	10.0	40.0		65.0	25.0	10.0	
High	37.5	37.5	25.0		37.4	31.3	31.3	
Marital status, *n* (%)				0.489				0.301
Married	63.6	18.2	18.2		36.3	18.2	45.5	
Single	31.7	35.4	32.9		44.3	30.4	25.3	
SES, *n* (%)				0.064				0.298
Low	0.0	20.0	80.0		40.0	20.0	40.0	
Middle	36.2	36.2	27.5		47.8	29.0	23.2	
High	50.0	22.2	27.8		27.8	33.3	38.9	
Stress, *n* (%)				0.375				0.877
Normal	40.0	40.0	20.0		43.3	26.7	30.0	
Mild	40.0	26.7	33.3		46.7	20.0	33.3	
Moderate	33.3	33.3	33.3		48.1	40.7	11.2	
Severe	29.4	23.5	47.1		29.4	29.4	41.2	
Extremely severe	66.7	33.3	0.0		66.7	0.0	33.3	
Appetite	31.68 (10.19)	32.63 (7.80)	33.11 (5.67)	0.766	31.40 (8.83)	34.46 (6.45)	31.92 (8.73)	0.299
LDL-C (mg/dl)	127.12 (40.14)	116.93 (25.42)	113.67 (32.56)	0.261	121.58 (34.47)	106.65 (28.75)^∗^	127.17 (34.58)^∗^	0.050
HDL (mg/dl)	48.15 (9.07)	46.43 (9.66)	47.56 (9.62)	0.766	48.90 (9.71)	46.08 (8.48)	46.40 (9.67)	0.404
Cholesterol (mg/dl)	195.62 (41.23)	186.13 (26.78)	181.56 (37.96)	0.298	190.28 (37.62)	172.58 (30.43)^∗^	197.33 (36.04)^∗^	0.022
TG (mg/dl)	101.74 (35.29)	113.83 (47.24)	101.67 (40.92)	0.422	106.30 (43.21)	99.23 (35.94)	111.48 (43.42)	0.569
AIP	-0.05 (0.21)	0.01 (0.25)	-0.05 (0.20)	0.518	-0.05 (0.24)	-0.04 (0.18)	0.00 (0.24)	0.672
Glucose (mg/dl)	90.94 (12.21)	91.43 (11.78)	92.44 (8.88)	0.871	91.60 (11.62)	92.31 (9.36)	90.68 (12.13)	0.873
Insulin, U/mL	13.55 (7.98, 25.73)	16.20 (9.78, 25.70)	14.60 (9.80, 21.00)	0.882	15.25 (10.00, 23.98)	16.30 (9.78, 25, 80)	14.50 (8.75, 24.85)	0.728
HOMA-IR	3.41 (1.74, 5.66)	3.53 (2.15, 6.17)	3.42 (2.13, 5.03)	0.902	3.45 (2.24, 5.54)	3.65 (2.26, 5.80)	3.20 (1.80, 5.96)	0.868
QUICKI	0.33 (0.04)	0.32 (0.03)	0.32 (0.03)	0.776	0.32 (0.03)	0.32 (0.03)	0.33 (0.03)	0.764
SBP (mmHg)	116.15 (18.01)	114.43 (12.15)	112.44 (15.80)	0.582	115.03 (17.14)	112.15 (13.08)	116.04 (15.46)	0.565
DBP (mmHg)	75.35 (10.32)	79.67 (10.51)	75.67 (14.65)	0.270	77.20 (11.06)	73 81 (9.30)	79.52 (14.84)	0.196
MetS (%)	36.0	40.0	24.0	0.758	52.0	16.0	32.0	0.804
MC4R (%)				0.044				0.645
CC	12.5	37.5	50.0		43.7	25.0	31.3	
CT	39.3	28.6	32.1		42.9	42.9	14.2	
TT	42.9	32.1	25.0		42.9	17.8	39.3	

Data are presented as mean (SD) or median (25 and 75 percentiles). ^∗^Analysis of variance for continuous variables and *χ*^2^ test for categorical variables. Abbreviations: BMI: body mass index; WC: waist circumference; SES: socioeconomic status; HOMA-IR: homeostasis model assessment of insulin resistance; LDL-C: low-density lipoprotein cholesterol; HDL: high-density lipoprotein; SBP: systolic blood pressure; DBP: diastolic blood pressure; TG: triglyceride; QUICKI: quantitative insulin sensitivity check index; AIP: atherogenic index of plasma; MC4R: melanocortin-4 receptor.

**Table 3:** Statistically significant direct and indirect pathways of the association of the MC4R rs17782313 polymorphism, diet, sociodemographic, and psychological variables with serum glycemic levels and lipid profile and MetS among obese individuals.

Model path	Standardized estimate^∗^	SE	*P*
Model 1			
*Direct effects*			
Age ⟶ GI	-0.105	0.045	0.019
Appetite ⟶ GL	1.795	0.533	≤0.01
Sex⟶ triglyceride	-30.589	9.604	≤0.01
Triglyceride ⟶ LDL-C	-0.200	0.001	≤0.01
HDL⟶LDL-C	-0.999	0.005	≤0.01
Cholesterol ⟶ LDL-C	1.001	0.001	≤0.01
Triglyceride ⟶ HDL	-0.068	0.011	≤0.01
Cholesterol ⟶ HDL	0.115	0.021	≤0.01
MC4R⟶HDL	-1.880	0.863	0.029
Age⟶ cholesterol	1.008	0.346	≤0.01
*Indirect effects via GI and GL*			
Sex ⟶LDL-C	3.970	1.448	≤0.01
MC4R⟶LDL-C	6.589	3.247	0.042
Age ⟶LDL-C	0.878	0.304	≤0.01
Sex ⟶HDL	2.203	0.764	≤0.01
Age ⟶HDL	0.129	0.049	≤0.01
Model 2			
*Direct effects*			
Age ⟶ GI	-0.103	0.045	0.022
Appetite ⟶ GL	1.797	0.534	≤0.01
Sex ⟶ glucose	-0.028	0.013	0.029
Age⟶ glucose	0.003	0.001	≤0.01
Age⟶ insulin	0.008	0.003	≤0.01
Model 3			
*Direct effects*			
MC4R⟶MetS	0.010	0.005	0.023
Sex⟶MetS	-0.605	0.268	0.024
Age⟶MetS	0.053	0.018	≤0.01

Abbreviations: GI: glycemic index; GL: glycemic load; LDL: low-density lipoprotein; HDL: high-density lipoprotein; MetS: metabolic syndrome; MC4R: melanocortin-4 receptor; SE: standard error of the estimate. All standardized path coefficients shown were significant (*P* < 0.05). ^∗^Standardized path coefficients.

**Table 4:** Goodness of fit indices for models.

Model	DF	*χ* ^2^	*χ* ^2^/DF	RMSEA	SRMR	CFI
1	24	28.974	1.207	0.038 (0.000-0.082)	0.050	0.996
2	11	11.334	1.030	0.015 (0.000-0.091)	0.031	0.987
3	4	4.813	1.203	0.037 (0.000-0.133)		0.994

*χ* ^2^: chi-square value; DF: degrees of freedom; RMSEA: root mean square error of approximation; SRMR: standardized root mean square residual; CFI: comparative fit index. (1) The final model with the best fit according to the values of several fit indices for the associations of genetic, sociodemographic, psychological parameters, and diet with lipid profile. (2) The final model with the best fit according to the values of several fit indices for the association of genetic, sociodemographic, psychological parameters, and diet with serum glycemic levels. (3) The final model with the best fit according to the values of several fit indices for the association of genetic, sociodemographic, psychological parameters, and diet with metabolic syndrome.

**Table 5:** Total effects of genetic, sociodemographic, and psychological parameters and diet on metabolic syndrome among obese adults using SEM.

Model 3	Total		
	Standardized estimate^∗^	SE	*P* value
GI ⟶ MetS	-0.002	0.002	0.117
GL ⟶ MetS	0.000	0.001	0.449
MC4R ⟶ MetS	0.010	0.005	0.023
Age ⟶ MetS	0.053	0.018	≤0.01
Sex ⟶ MetS	-0.605	0.268	0.012
Sex⟶ GL	-27.368	12.681	0.015
Age ⟶ GL	-0.073	0.721	0.459
PA ⟶ GL	-0.841	7.187	0.453
SES ⟶ GL	-0.600	2.462	0.403
Stress ⟶ GL	0.062	0.606	0.459
Appetite ⟶ GL	1.178	0.562	0.018
MC4R ⟶ GL	-1.235	0.188	≤0.01
Sex ⟶ GI	-1.391	9.004	0.433
Age ⟶ GI	-0.478	0.527	0.382
PA ⟶ GI	-1.089	7.271	0.440
SES ⟶ GI	-0.287	1.833	0.438
Stress ⟶ GI	0.310	0.451	0.246
Appetite ⟶ GI	0.772	0.478	0.053
MC4R ⟶ GI	1.577	0.173	≤0.01

Abbreviations: GI: glycemic index; GL: glycemic load; SES: socioeconomic status; MetS: metabolic syndrome; PA: physical activity; MC4R: melanocortin-4 receptor; SE: standard error of the estimate. ^∗^Standardized path coefficients ^**£**^Total effect is defined as the sum of direct and indirect effects.

## Data Availability

Data are available with a reasonable request from the corresponding author.
